# Characterizing Micro-scale Disparities in Childhood Obesity: Examining the Influence of Multilevel Factors on 4-Year Changes in BMI, Healthy Eating, and Physical Activity, Among a Cohort of Children Residing in Disadvantaged Urban Enclaves

**DOI:** 10.3389/fpubh.2019.00301

**Published:** 2019-10-22

**Authors:** Deborah Salvo, Nalini Ranjit, Aida Nielsen, Nika Akhavan, Alexandra van den Berg

**Affiliations:** ^1^Prevention Research Center, Brown School, Washington University in St. Louis, St. Louis, MO, United States; ^2^Michael & Susan Dell Center for Healthy Living, School of Public Health in Austin, The University Health Science Center in Houston, Austin, TX, United States

**Keywords:** childhood obesity, urban enclaves, BMI, healthy eating, physical activity, socio-ecologic model

## Abstract

The childhood obesity epidemic in the United States disproportionately affects minority, low-income populations. Hispanics have one of the highest childhood obesity rates, and are the fastest growing population subgroup in the country. Past research has examined disparities in the occurrence of obesity, healthy eating, and physical activity at a macro-geographic level, with less emphasis on examining the multilevel, micro-scale determinants of childhood obesity in disadvantaged urban ethnic enclaves. The aim of our study was to identify child-, parental-, familial-, community-, and neighborhood-level factors associated with differences in 4-year changes in BMI, healthy eating, and physical activity, among children residing in low-income, predominantly Hispanic urban enclaves in Austin, Texas. This analysis used data from the *Go Austin! Vamos Austin!* (GAVA) Evaluation study, a cohort with 4 years of follow-up from 313 child-caregiver dyads. The dependent variables were change categories denoting 4-year increase, decrease, or no change in Body Mass Index (BMI) percentile, fruit and vegetable intake, and physical activity, among child participants. The independent variables were factors at multiple levels of the socio-ecological model: child, parental, familial, community, and environmental. Multinomial logistic regression models were used to estimate the odds of children being in the “increasing” or “decreasing” categories for the three dependent variables (vs. “no change”), in association with the studied independent variables. The results showed that among children residing in this low-income, predominantly Hispanic urban enclave, weight gain prevention and weight loss have different determinants. We identified relevant micro-scale disparities, and micro-level factors of influence on child BMI and its related health behaviors, at all levels of the socio-ecological model. Our results revealed evidence, through the characterization of *positive deviance* cases (children for whom decreases in BMI, increases in fruit and vegetable intake, or increases in physical activity were observed) which could potentially help mitigate established unhealthy habits among high need populations. Factors associated with *positive deviance* for BMI (decreases in child BMI) included male child sex (OR: 0.33, 95% CI: 0.12–0.83) and living in a food-insecure household (OR: 0.24, 95% CI: 0.13–0.79). Our findings may inform the design of obesity prevention interventions in these types of disadvantaged urban Hispanic enclaves.

## Introduction

Childhood obesity has well-documented negative impacts on short- and long-term health outcomes, including an increased risk of becoming obese adults, and of developing several chronic diseases in later phases of life (type II diabetes, cardiovascular disease, some cancers), as well as immediate negative consequences for the child—like depression (1–4). In the United States (US) and globally, the rates of childhood obesity have dramatically increased over the past decades (5, 6). Approximately one in five US children or adolescents has obesity (7). These population-wide increases in childhood obesity have been characterized as an epidemic, which co-exists and interacts with other important global public health challenges: undernutrition and climate change (8). In the US, the obesity epidemic is known to disproportionately affect minority, low-income population subgroups (9). Increasing childhood obesity among Hispanics is of particular concern, given their high prevalence of obesity and associated chronic conditions, and the fact that they are the fastest growing population subgroup in the country (10).

A substantial body of evidence demonstrates that multiple factors influence weight status, as well as eating and physical activity behaviors among children (11, 12). These range from biological aspects of the child (e.g., their sex or age), to individual psychosocial aspects (e.g., self-efficacy, knowledge, or attitudes), to inter-personal factors (e.g., family support and preferences), to a broader social environment (e.g., cultural norms, crime levels, area-level racial/ethnic distribution), to aspects of the physical environment (availability and accessibility of grocery stores, parks and recreation facilities). Socio-ecological models propose that individual health and its associated behaviors are a result of the influence of all these levels, which interact with each other to influence the individual's behaviors and health outcomes (11, 13). Although there is convincing evidence of the influence of multilevel factors on childhood obesity, physical activity, and healthy eating, there are large intra- and intergroup variations in the significance, direction and strength of observed associations. These variations are inevitable given the number, complexity and variety of interactions among socio-ecological levels of influence, which are often patterned by geographic, cultural and sociodemographic contexts. Indeed, a case may be made for developing locally varying social ecologic models (14).

To date, several studies have examined disparities in the occurrence of obesity, healthy eating, and physical activity, across different socioeconomic and ethnic groups at a macro-geographic level (15–20). For instance, the 500 Cities Project data show that low-income census tracts with high proportions of minority populations have higher rates of obesity than their high-income, predominantly white geographic counterparts (21). Thus, children residing in geographic units (i.e., neighborhoods) consisting of primarily low-income, Hispanic residents, are typically uniformly characterized as being “at higher risk” for obesity, unhealthy eating, insufficient physical activity, and their associated co-morbidities. However, these macro-scale (neighborhood level) disparities are only one piece of the puzzle. Although the occurrence of the condition (obesity) is significantly higher in these geographic settings when compared to national averages, there is also substantial *intra-group variation* in the rates occurrence of obesity and its key behavioral determinants among residents of these so-called “disadvantaged urban ethnic enclaves.” That is, in addition to macro-scale disparities (across neighborhoods), there are also micro-scale disparities (within neighborhoods) in childhood obesity and its related health behaviors. To date, little research has been conducted to identify the multilevel determinants of such micro-scale disparities, in high-risk macro-scale areas (e.g., low-income, predominantly Hispanic urban enclaves).

The aim of our study was to conduct an exploratory secondary data analysis to identify child-, parental-, familial-, social-, and neighborhood-level factors associated with differences in 4-year change status for BMI, healthy eating, and physical activity, among children residing in low-income, predominantly Hispanic urban enclaves in Austin, Texas, USA. Nearly 40% of Texas residents are Hispanic, and the state is a majority-minority state, with most Hispanics being either of Mexican origin or of Mexican descent (22). Insights into micro-level determinants of child obesity have relevance statewide as well as nationwide, given the rapid growth of the Hispanic population across the US.

## Methods

### Study Design and Sample

This exploratory secondary data analysis used baseline and follow-up cohort data from the *Go Austin! Vamos Austin! Evaluation (GAVA) Evaluation Study*, obtained from residents of selected zip codes located in Austin's Eastern Crescent (23). The Eastern Crescent of Austin is predominantly Hispanic (and mostly populated by people of Mexican origin or descent), with several of the zip codes in the bottom 10 percent of all zip codes in Texas when classified by income. This area has a notably deficient built environment and infrastructure for PA and healthy eating, limited transportation mobility, and high concentrations of child obesity (24, 25). It has been identified as a priority area for developing, testing and implementing community-driven strategies for healthy eating and physical activity. The parent evaluation study was conducted within the context of a natural experiment to assess the GAVA intervention, a community-based led coalition that has promoted several community-wide strategies to improve healthy living in the Dove Springs area of the Eastern Crescent since 2013 (24). Preliminary data suggest some favorable changes in adult obesity and health behaviors in the first 5 years, but there is little evidence of positive overall change in child-level outcomes (unpublished data). This was expected, given that the intervention was not child-focused.

Extensive information on the design, sampling, and data collection of the parent study is available elsewhere (23). Briefly, the parent study that evaluated GAVA recruited and followed a cohort of 313 child-caregiver dyads, consisting of children enrolled in Kindergarten at baseline, and their self-identified primary adult caregivers. The sample was balanced to include an equal proportion of child-caregiver dyads residing in Dove Springs and in other areas of the Eastern Crescent that were not the focus of this natural experiment. To ensure a balanced spatial sample of the study area, the child-caregiver dyads were recruited from each of the 10 elementary schools located in the recruitment area. Schools were used as strategic locations to enroll a spatially balanced sample across the study area, defined by the zip codes of interest (Austin Eastern Crescent). In the parent study, a recruitment target of 30 dyads per school was set. The first 30 parents/caregivers and children dyads that approached us for participation were included in the study. Strategies for recruitment included flyers throughout the community (within the schools, in community centers, parks, etc.), and setting up tables during parent-teacher school nights in the 10 public schools serving the study area. One baseline assessment was conducted in 2013, and four follow-up yearly assessments were done from 2014 to 2017.

Aside from residence in one of the surveyed zip codes and attendance at one of the elementary schools, eligibility criteria required an adult (≥18 years of age) primary caregiver to provide written consent for the child-caregiver dyad to participate in the study, and verbal assent from the child. The primary caregiver had to be an English or Spanish speaker, and not enrolled in any other research study upon recruitment.

Recruitment and baseline assessment were done at the start of the school year within each of the schools by trained study staff. Follow-up data from caregivers were obtained either at the school or, where required, at the home of the participants. At each measurement period, caregivers were administered a survey questionnaire with questions pertaining to the child's behaviors, their own behaviors, and their perceptions of neighborhood resource availability. In addition, objectively measured height and weight data were obtained from both the child and the caregiver. The GAVA Evaluation study was approved by the Institutional Review Board of the University of Texas Health Science Center at Houston School of Public Health (HSC-SPH-13-0107).

### Measures

#### Dependent Variables

##### Change in child BMI

Categories of change of child BMI, from baseline to follow-up, were used as the primary dependent variable for this analysis. Child BMI was derived using objectively measured weight and height, using fixed stadiometers and calibrated scales, with standardized measurement protocols administered by trained data collectors (26). More information on the measurement protocol is available elsewhere (23). For each child, computed BMI scores were used to determine their BMI percentile at each assessment time-point, using the Centers for Disease Control and Prevention sex- and age-specific BMI percentiles for children ages 2–20 years (27, 28). Next, we estimated baseline to follow-up change in BMI percentile. This was done by subtracting the baseline BMI percentile (2013) value from the BMI percentile value of the longest available follow-up BMI data for each child (2014, 2015, or 2017). For the majority (>75%) of study participants, 4-Year (2013–2017) follow-up BMI data were available. Finally, participants were categorized as being in either the “increasing BMI,” “decreasing BMI,” or “no change in BMI” categories. Children were classified as being in the “increasing” or “decreasing” groups if the difference between their follow-up BMI percentile and their baseline BMI percentile was greater or equal to ±10 percentile points. Otherwise, participants were categorized as being in the “no change in BMI category.”

##### Changes in child health behaviors

In addition to examining baseline to follow-up change in BMI, we built analogous change variables to categorize child participants depending on whether they increased, decreased, or did not modify their weekly frequency of fruit and vegetable intake, and of participation in physical activity, respectively. Both of these secondary dependent variables were derived using questionnaire data from the parent GAVA Evaluation cohort study (23). In each assessment time point, primary caregivers responded to “How many days per week does your child eat five servings of fruit and vegetables?,” and “How many days per week is your child physically active at least 30 min?” For both items, response options included “less than 1 day per week,” “2–3 days per week,” “4–5 days per week,” and “more than 5 days per week,” and were scored to represent weekly frequency of these behaviors. Next, we calculated the difference between the weekly follow-up frequency of each of these behaviors and the baseline weekly frequency. Child participants with the same caregiver-reported weekly frequency at baseline and follow-up were categorized in the “no change” group, for each respective health behavior variable. Meanwhile, children having had any increase or decrease in frequency for each of these health behaviors were categorized as being in the “increasing” or “decreasing” groups for fruit and vegetable intake or physical activity participation frequency, respectively (i.e., a minimum difference of ±1 day per week was deemed as being an increase or decrease in weekly frequency).

#### Independent Variables

A series of baseline (2013) variables at multiple levels of the ecological model were included in this study as independent variables. All but one (parental BMI, which was derived from objectively measured height and weight) were based on the GAVA Evaluation baseline survey (self-reported instrument administered to primary caregivers) (23).

##### Child-level measures

Two demographic variables about the child, both biological, were included in this analysis: child sex and child age at baseline.

##### Parental-level measures

A series of parental measures were included in this study. These included variables about (a) the weight status (BMI, calculated using objectively measured height and weight at baseline), self-reported frequency of fruit and vegetable intake, and self-reported frequency of participation in physical activity, and (b) demographic characteristics of the primary caregiver.

Caregiver fruit and vegetable intake was assessed using two survey items: “What is the total amount of fruit you eat each day?” and “What is the total amount of vegetables you eat each day?” These questions were sourced from the Fruit & Vegetable Screeners in the Eating at America's Table Study (27). The response options were five categories in 0.5 cup increments, ranging from 0 to >2.5 cups per day. Total cups of fruits plus vegetables were calculated (theoretical range of 0 to >5 cups per day). For this analysis, the variable was dichotomized (≥3 cups per day, vs. <3 cups per day).

Caregiver minutes per week of moderate- to vigorous-intensity physical activity was assessed at baseline using a modified version of the self-administered past week physical activity questionnaire. For analysis, this variable was dichotomized, based on the Physical Activity Guidelines for Americans (≥150 min per week, vs. <150 min per week) (29).

The primary caregiver demographic variables included in this analysis were sex (male/female), age at baseline (years), and race/ethnicity (dichotomized as Hispanic vs. non-Hispanic).

##### Family-level measures

Conceptually, we considered familial-level characteristics those that tend to affect the full family's socioeconomic status, composition, or dynamics (in contrast to being only relevant to the primary caregiver).

To assess family socioeconomic status we used self-reported measures for the educational attainment of the primary caregiver (dichotomized for analysis as high school or more, vs. less than high school), annual household income (dichotomized for analysis as >$35,000 USD per year vs. ≤$35,000 USD per year), food insecurity (whether the family sometimes or always ran out of food before the next paycheck), and whether the family was a beneficiary/user of government assistance programs at baseline (dichotomized as ≥1 or more programs vs. 0 programs; includes free or reduced lunch school program, food stamps/SNAP, TANF, WIC, Medicaid, and CHIP).

Measures of family composition and dynamics included single item measures of family size (number of adults in the household, number of children in the household), acculturation (language spoken at home), and marital status of the primary caregiver. For analysis, these variables were dichotomized: single adult household (vs. ≥2 or more adults in the household), many children household (≥3 or more children, vs. <3 children), Spanish only household (vs. only English or mix of Spanish and English spoken at home), and parents or caregivers are married or live together (vs. divorced, separated, widowed, or single parents/caregivers).

##### Perceived community social norms (social environment)

Two survey items were used to determine the baseline perceptions of primary caregivers of how healthy their community social norms is: “In your neighborhood, how many people do you know that think healthy eating is important?” (4-point Likert scale), and “In your neighborhood, a lot of people walk” (3-point Likert scale). Both variables were dichotomized and reverse-coded for analysis, to reflect if “neighbors do not value healthy eating” (vs. they do value healthy eating), and if “most neighbors do not walk” (vs. most neighbors walk).

##### Perceived food and built neighborhood environment

Neighborhood environment measures included in our analysis were those pertaining to the perceived access to healthy foods in the neighborhood, perceived quality of the neighborhood built environment, and perceived safety of the neighborhood for children to be physically active in it.

Perceived access to healthy foods in the neighborhood was assessed through three survey items, which inquired on whether the primary caregiver experiences issues when purchasing fruits and vegetables at local stores for their family. The potential issues included the fruits and vegetables available being of low quality, the selection of available fruits and vegetables for purchase being poor, and the fruits and vegetables available being too expensive. For each of these, answer options for the respondent were yes or no (i.e., respondents either confirmed or denied each as an issue).

Perceptions of the quality and safety of the neighborhood built environment were determined using the following survey items: “What is the general quality of your neighborhood's sidewalks, streets and open spaces?” (response options were assessed using a 4-point Likert scale ranging from “poor” to “excellent), and “In general, do you feel like your neighborhood is a safe place for your child to play outside?” (response options were assessed using a 3-Point agreement-based Likert scale). For this study, both variables were re-coded as binary, denoting the neighborhood built environment being of good quality (vs. not good quality) and the neighborhood being unsafe for children to play outside (vs. the neighborhood being safe).

### Statistical Analysis

All analyses were conducted using SAS software, version 9.4 (SAS Institute, Cary, North Carolina). Descriptive statistics were calculated to characterize the study sample, and included means and standard deviations, and frequencies and percentages.

Next, a series of multinomial logistic regression models were run to determine whether the multiple independent variables of interest were associated with children's *change categories* for the dependent variables. For the three dependent variables of interest (change in BMI, change in fruit and vegetable intake, and change in physical activity—all for the child), the “no change” category was used as the referent in the models. This meant determining the effect of socio-ecological factors on the odds of being in the “decreasing” and/or in the “increasing,” as separate outcome categories. Because the intra-cluster correlation coefficient (ICC) for school-level clustering of the independent variable was <0.01, single-level models were run.

The modeling process was conducted in three steps. First, a series of “single independent variable” models were run. For each dependent variable, separate models were run for each of the independent variable listed in the measures section above, corresponding to factors at multiple levels of the ecologic model that we hypothesized may influence longitudinal changes in BMI percentile, fruit and vegetable intake, and physical activity levels among children. The estimates of these first step models were only adjusted for time of follow-up and exposure status (yes/no, based on residential zip code in the Dove Springs neighborhood) to the community-led strategies to improve the food and built environment. Next, all independent variables found to have a significant association with the respective dependent variable at the *p* < 0.10 level, were included in a second adjusted model. In this step, the two child-level independent variables (child sex and age at baseline) and follow-up time were always forced into the models, independent of statistical significance in the first modeling step (unadjusted models). Exposure status to the community-led environmental strategies to promote healthy eating and physical activity was not included as a covariate in the fully adjusted models, as it was not associated with any of the three dependent variables at the *p* < 0.10 level (consistent with earlier findings of no intervention effect on child outcomes). The third and final step of this analysis corresponded to model diagnostics, to ensure that multicollinearity was not an issue. Multicollinearity was assessed using VIF values (>10 considered problematic), and by examining the covariance matrix and corresponding eigenvalues for each variable, to discard excessive shared variance amongst two or more covariates in the model. Odds Ratios (OR) and 95% Confidence Intervals (CI) were calculated for all modeling results. Statistical significance for the final adjusted model per dependent variable was set at an alpha level of *p* < 0.05.

## Results

Of the 313 child-caregiver dyads recruited at baseline in the year 2013, 286 had complete data for this analysis (retention rate of 91.4%) (i.e., provided at least 1 year of follow-up data). Because less than 10% of the sample had missing data, and given that no significant differences were observed when comparing the characteristics of participants with complete vs. incomplete data, the results herein reported correspond to a complete case analysis. The final analytic sample size for this exploratory secondary data analysis was of 286 child-parent dyads.

The cohort of children had a fairly even balance by sex (47.6% female), and most children were under 6 years of age at time of baseline assessment (76.2%). At baseline, 26.9% of the children had obesity (≥95th BMI percentile for their age), while 32.3% did so at follow-up. The average follow-up time was of 3.7 years (*SD* = 0.8; median and 75th percentile = 4 years). In terms of caregiver and familial characteristics, 53.1% of the children's caregivers had obesity at baseline, 59.1% were physically active, and 13.3% ate more than 3 cups of fruits or vegetables per day. Most caregivers were aged 31–45 years (52.1%), and most families had low socioeconomic status per different indicators (education, household income, food insecurity, beneficiaries of government assistance). Full descriptive characteristics of the study sample are shown in Table 1.

**Table 1:** Child, parental, familial, community and environmental characteristics of the *Go Austin! Vamos Austin!* Cohort at baseline (2013; *n* = 286).

**Ecological characteristic**	***n***	**%**
**Child obesity and demographics**		
Obese (≥95th BMI percentile)	77	26.9
Child age is ≥6 years	68	23.8
Female	136	47.6
**Caregiver obesity and related behaviors^a^**		
Obese (BMI ≥ 30)	152	53.1
F&V^b^ Intake > 3 cups per day	38	13.3
Physically active (≥150 min/week of MVPA^c^)	169	59.1
**Caregiver demographics^a^**		
Age		
18–30 years	117	40.9
31–45 years	149	52.1
>45 years	20	7.0
Female	260	90.9
Hispanic race/ethnicity	247	86.4
**Family socioeconomic status**		
Primary caregiver has a high school or higher education level	153	53.5
Household income (yearly)		
Up to $15,000 USD	137	47.9
$15,001–35,000 USD	120	42.0
>$35,000 USD	31	10.1
Food insecure household^d^	165	57.7
Beneficiary of government assistance programs^e^	201	70.3
**Family composition and dynamics**		
Married parents^f^	181	63.3
Singe adult household	62	21.7
≥3 children household	150	52.4
Only Spanish spoken at home	122	42.7
**Perceived community social norms (social environment)**		
Neighbors do not value healthy eating	100	35.0
Neighbors do not walk	253	88.5
**Perceived food and built neighborhood environment**		
It is difficult to find fruits and vegetables in local stores that are…		
Of good quality	50	17.5
Of good variety (good selection)	63	22.0
Inexpensive	87	30.4
The built environment for PA in the neighborhood is of good quality^g^	126	44.1
The neighborhood is unsafe for children to play outside	253	88.5

a *Refers to primary caregiver only*.

b *Fruits and vegetables*.

c *Moderate- to vigorous-intensity physical activity*.

d *Determined by caregiver reporting sometimes or almost always running out of food at home by the end of the week*.

e *Determined by primary caregiver reporting regularly using at least one benefits/assistance program*.

f *Based on reported marital status of primary caregiver. Married category includes those reporting living with a partner*.

g *Includes sidewalks, streets, and open spaces*.

Table 2 presents the mean and standard deviation values of child BMI percentile, child fruit and vegetable intake, and child physical activity frequency, at baseline and follow-up. Among the total sample, the average absolute and relative changes in BMI percentile score from baseline to follow-up were positive. Consistently, average absolute and relative changes in healthy behaviors (fruit and vegetable intake and physical activity) were negative for the full sample. Among the sample of children, 30.0% had an increase in BMI, and 15.4% had a decrease in BMI.

**Table 2:** Baseline and follow-up status, and longitudinal changes in Body Mass Index (BMI) and health behaviors among children enrolled in the *Go Austin! Vamos Austin!* Cohort study (2013–2017).

**BMI or health behavior variable**	**Baseline** **mean ± SD**	**Follow-Up^a^mean ± SD**	**Absolute Change** **mean ± SD**	**Relative (%) Change^b^mean ± SD**
**Child BMI percentile**				
Total (*n* = 286, 100%)	66.3 ± 31.0	70.2 ± 30.7	4.4 ± 22.6	6.7 ± 34.2
No change group (*n* = 156, 54.5%)	79.1 ± 29.0	79.2 ± 29.2	0.2 ± 4.1	0.3 ± 5.1
Decreasing BMI group (*n* = 44, 15.4%)	60.2 ± 24.1	32.9 ± 23.2	−27.3 ± 19.7	−49.2 ± 27.3
Increasing BMI group (*n* = 86, 30.0%)	44 ± 24.3	74.0 ± 21.2	29.1 ± 17.7	66.2 ± 41.0
**Daily F&V^c^ intake**				
Total (*n* = 286)	1.6 ± 0.8	1.5 ± 0.9	−0.05 ± 1.0	−0.4 ± 62.7
No change group (*n* = 133, 46.5%)	1.5 ± 0.7	1.5 ± 0.7	0.0 ± 0.0	0.0 ± 0.0
Decreasing F&V Intake (*n* = 83, 29.0%)	2.1 ± 0.7	0.9 ± 0.7	−1.2 ± 0.5	−63.3 ± 25.2
Increasing F&V intake (*n* = 74, 24.5%)	1.0 ± 0.7	2.3 ± 0.7	1.3 ± 0.5	98.9 ± 53.4
**Days/week of PA^d^**				
Total (*n* = 286)	2.0 ± 0.9	1.7 ± 0.9	−0.4 ± 1.0	−14.9 ± 53.2
No change group (*n* = 114, 40.0%)	1.8 ± 0.9	1.8 ± 0.9	0.0 ± 0.0	0.0 ± 0.0
Decreasing PA (*n* = 128, 44.9%)	2.5 ± 0.6	1.2 ± 0.7	−1.3 ± 0.6	−55.6 ± 22.3
Increasing PA (*n* = 44, 15.4%)	1.1 ± 0.8	2.5 ± 0.6	1.3 ± 0.6	91.4 ± 50.1

a *Longest follow-up period with complete data available per participant was used (ranges from 1 to 4 years, being 4 years for >75% of the sample, 3 years for 20% of the sample, and 1–2 years for <5% of the sample)*.

b Calculated as [(value at follow-up – value at baseline)/value at baseline] *100.

c *Fruits and vegetables (number of days per week when the child eats 5 or more servings of fruits and vegetables)*.

d *Physical activity (number of days per week when the child is physically active for 30 min or more)*.

Consistent patterns were observed for the two examined health behaviors (Table 2). Among the children cohort, 46.5% had a stable (no change) fruit and vegetable intake from baseline to follow-up, 29% had a decrease in their fruit and vegetable intake, and 24.5% had an increase in intake. In terms of physical activity frequency, 40.0% percent of the children followed maintained their baseline levels of weekly participation in physical activity from baseline, 44.9% decreased their physical activity, and 15.4% showed increases in this healthy behavior.

### Effects of Multilevel Factors on Children's Increases or Decreases in BMI

Table 3 shows the results of the results of the unadjusted and adjusted estimates of the multinomial logistic models testing the associations of the independent variables on BMI change categories.

**Table 3:** Associations between baseline ecological characteristics and child Body Mass Index (BMI) change status from baseline to follow-up (referent is “no change” group); *Go Austin! Vamos Austin!* cohort (2013–2017; *n* = 286).

**Independent variable**	**Model 1^a^**		**Model 2^b^**	
	**Decreasing BMI group**	**Increasing BMI group**	**Decreasing BMI group**	**Increasing BMI group**
	**OR (95% CI)**	**OR (95% CI)**	**OR (95% CI)**	**OR (95% CI)**
**Child Demographics**				
Age	1.52 (0.45, 3.50)	0.94 (0.36, 1.57)	0.81 (0.31, 2.69)	0.55 (0.20, 1.82)
Sex (ref = male)	0.43 (0.29, 0.87)^*^	1.75 (0.93, 3.02)	0.33 (0.12, 0.83)^*^	1.48 (0.74, 3.42)
**Caregiver health^c^**				
Obese (BMI ≥ 30)	0.72 (0.33, 1.14)	0.77 (0.54, 1.38)		
F&V^d^ Intake > 3 cups per day	1.07 (0.36, 3.02)	0.70 (0.29, 1.78)		
Physically active^e^	1.31 (0.59, 2.61)	**0.64 (0.28, 1.02)**	1.66 (0.51, 5.49)	0.67 (0.42, 0.97)^*^
**Caregiver demographics^c^**				
Age	0.98 (0.97, 1.01)	0.98 (0.98, 1.01)		
Sex (ref = male)	1.02 (0.22, 3.56)	1.06 (0.38, 3.60)		
Hispanic (ref = other race/ethnicity)	0.72 (0.30, 1.65)	1.62 (0.61, 4.22)		
**Family socioeconomic status**				
≥High school education (ref = less than high school)^f^	1.27 (0.54, 2.11)	1.12 (0.45, 1.89)		
Household income ≥$35,000/yr (ref = lower than $35,000)	3.03 (1.24, 8.10)^*^	0.96 (0.36, 3.08)	1.73 (0.31, 9.54)	0.29 (0.11, 1.79)
Food insecure household^g^	0.42 (0.24, 0.76)^*^	0.58 (0.33, 1.20)	0.24 (0.13, 0.79)^*^	0.68 (0.30, 1.74)
Receives benefits^h^	**0.54 (0.21, 1.01)**	**0.59 (0.32, 1.01)**	0.82 (0.16, 3.10)	0.34 (0.13, 0.90)^*^
**Family composition/dynamics**				
Married parents^i^	1.01 (0.47, 2.29)	2.02 (1.02, 3.90)^*^	0.73 (0.15, 2.3)	1.53 (0.59, 3.64)
Singe adult household	1.34 (0.63, 2.91)	0.46 (0.17, 1.22)		
≥3 children household	0.89 (0.38, 1.72)	1.12 (0.60, 1.91)		
Only Spanish spoken at home	0.91 (0.36, 1.79)	0.60 (0.50, 1.14)		
**Perceived community social norms (social environment)**				
Neighbors…Do not value healthy eating	0.67 (0.29, 2.01)	2.03 (1.11, 4.02)^*^	0.64 (0.17, 1.90)	2.10 (1.14, 4.65)^*^
Do not walk	0.73 (0.44, 1.82)	0.64 (0.27, 1.21)		
**Perceived food & built neighborhood environment**				
Local stores lack F&V^d^ of good quality (ref = no)	1.17 (0.45, 2.82)	0.47 (0.22, 1.20)		
Local stores lack a good selection of F&V^d^ (ref = no)	1.50 (0.56, 3.43)	1.44 (0.69, 2.83)		
F&V^d^ at local stores are expensive (ref = no)	1.21 (0.60, 2.60)	0.74 (0.38, 1.42)		
The built environment of the neighborhood is of good quality^j^ (ref = no)	1.04 (0.44, 2.15)	0.41 (0.23, 0.66)^*^	0.67 (0.33, 1.19)	0.44 (0.24, 0.89)^*^
The neighborhood is unsafe for children to play outside (ref = no)	0.98 (0.20, 3.15)	1.57 (0.57, 3.60)		

P < 0.10 shown in bold, p < 0.05 shown in bold with an asterisk*.

a *Adjusted for time from baseline to follow-up and exposure status (yes/no) to GAVA coalition strategies*.

b *Includes all significant variables from model 2 at the p < 0.10 alpha level. Child age and sex, and time-to-follow-up were forced into the model*.

c *Primary caregiver*.

d *Fruits and vegetables*.

e *Engages in 150 min of moderate- to vigorous-intensity physical activity or more per week*.

f *Based on primary caregiver's highest level of attained education*.

g *Determined by caregiver reporting sometimes or almost always running out of food at home by the end of the week*.

h *Determined by primary caregiver reporting regularly using at least one benefits/assistance program*.

i *Includes unmarried couples that live together*.

j *Includes sidewalks, streets and open spaces*.

In the models of unadjusted estimates, four variables (representing two of the studied ecological levels of influence: child demographics and familial socioeconomic status) were associated with children being in the “decreasing BMI group.” Being a female child (vs. male), living in a food insecure household, and being a member of a family that receives government assistance were all associated with lower odds of being in the “decreasing BMI group,” as compared to the “no change in BMI group.” In the model of fully-adjusted estimates, only female child sex (vs. male, OR: 0.33, 95% CI: 0.12, 0.83) and belonging to a food insecure household (OR: 0.24, 0.13, 0.79) remained significantly associated with lower odds of being in the “decreasing child BMI group.”

A different set of explanatory variables were associated with children's odds of being in the “increasing BMI” group. In the first model of unadjusted estimates, having a physically active primary caregiver (parental level), belonging to a family that receives government assistance, and living in a neighborhood perceived as having a high quality built environment (perceived built neighborhood environment level) were inversely associated with children's odds of being in the “increasing BMI” group, at an alpha level deemed appropriate for further exploration in the final adjusted model. Also in the first model of unadjusted estimates, two factors were associated with higher odds of being in the “increasing BMI” group: belonging to a family with parents that are married or live together (familial level), and belonging to a community that is perceived by the child's caregiver as not valuing healthy eating (community social norms level). In the fully-adjusted model, parental marital status was no longer statistically significantly related to children being in the “increasing BMI” group. Having a physically inactive primary caregiver (OR: 0.67, 95% CI: 0.42–0.97), belonging to a family that receives government assistance (OR: 0.34, 95% CI: 0.13–0.90), perceiving that the community does not value healthy eating (OR: 2.10, 95% CI: 1.1–4.7), and living in a neighborhood perceived to be of high quality built environment (OR: 0.44, 95% CI: 0.24–0.89) remained significantly associated with the odds of being in the “increasing BMI” among children.

### Effects of Multilevel Factors on Children's Increases or Decreases in Healthy Behaviors

The results of the multinomial logistic models testing the association of multilevel factors on children's increase or decrease (vs. no change) in fruit and vegetable intake and physical activity are shown in Tables 4, 5, respectively. Generally, fewer factors were found to be consistently associated with increases and decreases in healthy behaviors, relative to what was observed in the “changes in BMI” models.

**Table 4:** Associations between baseline ecological characteristics and child fruit & vegetable (F&V) daily intake change status from baseline to follow-up (referent is “no change” group); *Go Austin! Vamos Austin!* cohort (2013–2017; *n* = 286).

**Independent variable**	**Model 1^a^**		**Model 2^b^**	
	**Decreasing F&V group**	**Increasing F&V group**	**Decreasing F&V group**	**Increasing F&V group**
	**OR (95% CI)**	**OR (95% CI)**	**OR (95% CI)**	**OR (95% CI)**
**Child demographics**				
Age	0.68 (0.30, 1.52)	2.43 (1.18, 5.21)^*^>	0.68 (0.29, 1.63)	2.64 (1.17, 5.93)^*^>
Sex (ref = male)	**0.52 (0.27, 1.04)**	0.76 (0.42, 1.37)	0.52 (0.28, 0.88)^*^>	0.73 (0.38, 1.44)
**Caregiver health^c^**				
Obese (BMI ≥ 30)	**1.71 (0.98, 3.20)**	1.19 (0.55, 2.21)	1.87 (1.13, 3.65)^*^>	1.60 (0.81, 3.10)
F&V Intake > 3 cups per day	1.46 (0.62, 3.3)	0.83 (0.34, 2.17)		
**Caregiver demographics^c^**				
Age	**1.14 (0.99, 1.12)**	1.02 (0.90, 1.08)	1.11 (1.08, 1.22)^*^>	1.03 (0.92, 1.07)
Sex (ref = male)	1.55 (0.41, 4.98)	0.88 (0.33, 2.85)		
Hispanic (ref = other race/ethnicity)	1.10 (0.36, 2.88)	0.51 (0.24, 1.23)		
**Family socioeconomic status**				
≥High school education (ref = less than high school)^d^	1.0 (0.5, 1.8)	1.21 (0.62, 2.24)		
Household income ≥$35,000/yr (ref = lower than $35,000)	0.7 (0.2, 1.8)	0.47 (0.18, 1.70)		
Food insecure household^e^	0.6 (0.3, 1.2)	1.13 (0.46, 2.12)		
Receives benefits^f^	1.5 (0.8, 3.0)	1.42 (0.67, 2.84)		
**Family composition/dynamics**				
Married parents^g^	1.42 (0.69, 2.20)	0.89 (0.45, 1.76)		
Singe adult household	0.70 (0.26, 1.48)	0.82 (0.44 (1.65)		
≥3 children household	1.14 (0.56, 2.01)	1.14 (0.49, 1.90)		
Only Spanish spoken at home	1.08 (0.53, 1.66)	1.20 (0.64, 2.28)		
**Perceived community social norms (social environment)**				
Neighbors don't value healthy eating	1.10 (0.47, 2.63)	1.12 (0.54, 2.41)		
**Perceived food neighborhood environment**				
Local stores lack F&V of good quality (ref = no)	1.62 (0.71, 3.39)	1.27 (0.56, 2.94)		
Local stores lack a good selection of F&V (ref = no)	0.83 (0.44, 1.79)	1.46 (0.72, 2.99)		
F&V at local stores are expensive (ref = no)	1.35 (0.67, 2.45)	0.66 (0.28, 1.42)		

P < 0.10 shown in bold, p < 0.05 shown in bold with an asterisk*.

a *Adjusted for time from baseline to follow-up and exposure status (yes/no) to GAVA coalition strategies*.

b *Includes all significant variables from model 2 at the p < 0.10 alpha level. Child age and sex, and time-to-follow-up were forced into the model*.

c *Primary caregiver*.

d *Based on primary caregiver's highest level of attained education*.

e *Determined by caregiver reporting sometimes or almost always running out of food at home by the end of the week*.

f *Determined by primary caregiver reporting regularly using at least one benefits/assistance program*.

g *Includes unmarried couples that live together*.

**Table 5:** Associations between baseline ecological characteristics and child weekly physical activity frequency (PA^a^) change status from baseline to follow-up (referent is “no change” group); *Go Austin! Vamos Austin!* cohort (2013–2017; *n* = 286).

**Independent variable**	**Model 1^b^**		**Model 2^c^**	
	**Decreasing PA^a^ group**	**Increasing PA^a^ group**	**Decreasing PA^a^ group**	**Increasing PA^a^ group**
	**OR (95% CI)**	**95% CI**	**OR**	**95% CI**
**Child demographics**				
Age	1.36 (0.58, 2.70)	0.78 (0.27, 1.91)	1.38 (0.71, 2.78)	0.72 (0.26, 2.02)
Sex (ref = male)	1.02 (0.64, 1.82)	1.90 (0.73, 3.56)	0.95 (0.56, 1.82)	2.11 (0.94, 5.02)
**Caregiver health^d^**				
Obese (BMI ≥ 30)	1.00 (0.6, 1.5)	1.77 (0.67, 3.64)		
Physically active^e^	**0.83 (0.39, 1.01)**	1.14 (0.45, 2.19)	**0.80 (0.39, 1.02)**	1.17 (0.45, 2.84)
**Caregiver demographics^d^**				
Age	0.98 (0.97, 1.06)	0.99 (0.92, 1.04)		
Sex (ref = male)	0.47 (0.18, 1.59)	0.65 (0.09, 2.75)		
Hispanic (ref = other race/ethnicity)	1.14 (0.40, 2.56)	1.34 (0.43, 4.21)		
**Family socioeconomic status**				
≥High school education (ref = less than high school)^f^	1.09 (0.57, 1.71)	0.71 (0.20, 1.53)		
Household income ≥$35,000/yr (ref = lower than $35,000)	1.27 (0.47, 3.52)	1.69 (0.54, 5.98)		
Food insecure household^g^	0.42 (0.24, 0.76)^*^	**0.53 (0.13, 1.02)**	0.54 (0.34, 0.87)^*^	2.10 (0.92, 5.01)
Receives benefits^h^	0.99 (0.50, 1.62)	0.68 (0.34, 1.46)		
**Family composition/dynamics**				
Married parents^i^	1.64 (0.89, 3.03)	1.11 (0.47, 2.40)		
Single adult household	0.67 (0.44, 1.41)	0.83 (0.18, 2.04)		
≥3 children household	1.23 (0.63, 1.87)	**1.68 (0.97, 3.75)**	0.95 (0.59, 1.87)	2.09 (1.04, 4.77)^*^
Only Spanish spoken at home	**1.61 (1.0, 2.7)**	1.60 (0.78, 3.50)	1.63 (0.89, 3.01)	1.59 (0.68, 3.71)
**Perceived community social norms (social environment)**				
Neighbors do not walk	1.01 (0.44, 1.99)	2.88 (0.52, 9.27)		
**Perceived built neighborhood environment**				
The built environment of the neighborhood is of good quality^j^ (ref = no)	1.20 (0.68, 2.20)	0.74 (0.37, 1.62)		
The neighborhood is unsafe for children to play outside (ref = no)	**1.98 (0.94, 4.86)**	0.91 (0.28, 2.93)	**2.23 (0.99, 5.96)**	1.06 (0.29, 3.60)

P < 0.10 shown in bold, p < 0.05 shown in bold with an asterisk*.

a * Number of days per week in which the child engages in at least 30 min of moderate- to vigorous-intensity physical activity, as reported by the primary caregiver*.

b *Adjusted for time from baseline to follow-up and exposure status (yes/no) to GAVA coalition strategies*.

c *Includes all significant variables from model 2 at the p < 0.10 alpha level. Child age and sex, and time-to-follow-up were forced into the model*.

d *Primary caregiver*.

e *Engages in 150 min of moderate- to vigorous-intensity physical activity or more per week*.

f *Based on primary caregiver's highest level of attained education*.

g *Determined by caregiver reporting sometimes or almost always running out of food at home by the end of the week*.

h *Determined by primary caregiver reporting regularly using at least one benefits/assistance program*.

i *Includes unmarried couples that live together*.

j *Includes sidewalks, streets, and open spaces*.

Three variables were significantly associated with children being in the “decreasing fruit and vegetable intake” group: female child sex (vs. male; adjusted OR: 0.52, 95% CI: 0.28–0.88), parental obesity (adjusted OR: 1.87, 95% CI: 1.13–3.65), and parental age in years (OR: 1.11, 95% CI: 1.08–1.22). Meanwhile, only one variable, child age, remained significantly associated with belonging to the “increasing fruit and vegetable intake.” Every unit increase in years of age was associated with 164% higher odds (95% CI: 1.17–5.93) of having increased fruit and vegetable intake from baseline to follow-up (vs. no change group).

As for the physical activity change models (Table 5), the final model with fully-adjusted estimates showed that belonging to a food insecure household was associated with 54% lower odds (95% CI: 0.34–0.87) of children having decreased their physical activity participation frequency from baseline to follow-up. After adjusting for covariates, marginal associations between having physically active parents (OR: 0.80, 95% CI: 0.39, 1.02) and living in a neighborhood perceived by parents as being unsafe for children to play outside (OR: 2.23, CI: 0.99, 5.96) with children being in the “decreasing physical activity” group were observed. After adjusting for covariates, only one factor was found to be significantly and directly associated with children having experienced an increase in their physical activity participation frequency from baseline to follow up: belonging to a family with three or more children (OR: 2.09, 95% CI: 1.04, 4.77).

## Discussion

This study explored potential multilevel determinants of changes over time in childhood BMI percentile and healthy behaviors (fruit and vegetable intake and physical activity) among a cohort of child-parent dyads residing in low income, predominantly Hispanic urban enclaves. Several factors acting at multiple levels of the ecological model were found to be associated children having either increased or decreased their BMI percentile over time. Although less robust than the BMI findings, the examination of how child, parental, familial, community, and neighborhood level factors may (or may not) influence change trajectories in these positive, healthy behaviors, revealed interesting findings.

Several findings are worth highlighting. The first and perhaps the most important takeaway, is the fact that the factors that may help prevent weight gain among children residing in low-income, minority settings, are not necessarily the same factors that can help promote weight loss in the same population. Some previous studies have assumed a linear relationship between BMI change and determinants of BMI, i.e., they assume that whatever is positively associated with increases in BMI is automatically related to potential decreases in BMI if used as an intervention tool (30–32). Our results contrast with such previous approaches, by showing virtually no overlapping weight gain risk factors as also being weight reduction promotion factors, suggest that such an interpretation oversimplifies the complexities of weight maintenance and weight loss (33).

In this study, we focused on a population subgroup that is pre-defined by their geographic area of residence as being at high risk for the adoption of unhealthy behaviors, and consequent progression into obesity and all of its associated co-morbidities in later life stages. By doing so, not only did we reveal the existence of micro-level disparities in childhood obesity occurring within this high-risk macro-level setting (as hypothesized), but also the fact that an important proportion of children fall under what can be categorized as *positive deviance* (34–36). Fifteen percent of the children experienced decreases in their BMI percentile score from baseline to follow-up, and a virtually equal proportion increased their participation in physical activity. Additionally, a quarter of the sample increased their fruit and vegetable daily intake. All of this occurred in a macro-environment (low-income, predominantly Hispanic urban enclaves) that supposes a high risk for childhood obesity, when compared with higher-income, white neighborhoods in the US. Our analysis identifying several ecological factors associated with belonging to the *positive deviance* group provides important evidence not only on how to prevent obesity among high-risk groups, but also on how to revert it. Our findings with regards to some factors associated with belonging in the *positive deviance* group (those who showed improvements in BMI status, healthy eating and physical activity, in spite of being located in a socioeconomically disadvantaged setting) are worth highlighting. These findings could be used to design and test culturally appropriate interventions to reduce health disparities in the Austin Eastern Crescent, and potentially in other similarly disadvantaged settings.

Our results are consistent with some recent studies stressing the importance of better understanding *positive deviance* in high-risk populations to better design and implement interventions for reverting childhood obesity disparities in disadvantaged settings (35, 36). However, our findings on the specific factors associated with *positive deviance* contrast with those of other recent studies of low-income, predominantly Hispanic communities. Foster et al. conducted a similar study in San Antonio, Texas, and identified low parental education, high parental self-efficacy, and predominant Mexican cultural identity (low acculturation) among parents, as factors associated with *positive deviance* for healthy weight trajectories in children aged 2–5 years of age (37). None of these factors were found to be associated with decreasing BMI in our study. Similarly, a mixed-methods study of Hispanic families in the lower Rio Grande Valley in Texas, found that family demographics, including income, education, and food assistance use, did not vary between groups of normal weight, overweight, and obese children (34). However, in our study, we observed that belonging to a food insecure household was inversely associated with the odds of being in the *positive deviance* (decreasing BMI) group.

The contrasting findings to other similar studies (both in terms on the focus on *positive deviance* and the communities under study—low-income, Hispanic groups) suggest that the drivers of healthy weight trajectories may be context-and community-specific. It is also important to consider the many cultural, sociodemographic, and biological differences across sub-population groups of Hispanics in the US. This study examined a population which is primarily of Mexican origin or descent. Further studies examining the multilevel correlates of positive deviance for BMI, healthy eating and physical activity trajectories among children are needed for other Hispanic communities.

With respect to the specific factors found to be associated with being in the “increasing” or “decreasing” BMI, fruit and vegetable intake, and physical activity groups among children of Austin's Eastern Crescent area, our findings show that family level food insecurity is a potentially strong influence impeding weight loss among children in these disadvantaged urban enclaves. However, it also appears to act as a protective factor against declines in physical activity participation. These findings are intriguing and perhaps counterintuitive, as typically, food insecurity is thought of as being related to hunger and unhealthy weight loss as a consequence (38). However, they are consistent with the notion of a food insecurity—obesity paradox (39). Likewise, food insecurity, through its links to hunger, has been previously posed as a determinant of low energy levels by those who suffer it (38, 40). However, we observed that children in food insecure households had lower odds of reducing their physical activity participation rates over time, which contradicts the “loss of energy to play” argument. On the other hand, food insecurity can be associated with low quality diets (38, 41). The influence of food insecurity on overall diet quality among children and families residing in high-risk urban enclaves requires further study.

Our study had several limitations that should be acknowledged. First, the small sample size (*n* = 286) may have limited our ability to detect other correlates of BMI change categories for children. The original parent study was designed and powered for a different purpose than this exploratory secondary data analysis (the main objective of the parent study was to detect differences in BMI when comparing participants residing in intervention vs. comparison zip codes). Second, the recruitment procedures of the original parent study (setting a cap of enrollment of 30 dyads per school in the target area, first-come-first-serve), may have led to selection bias in the sample. Third, most independent variables were dichotomized for analysis. This may have led to undetected associations across extreme categories, or of non-linear patterns. Future, larger studies are required to verify and expand our findings. Fourth, the survey questions, including those used to assess the two health behaviors of interest, and some of the independent variables, are fairly limited. The single item used to measure physical activity among children was restricted to frequency of participation in physical activities lasting at least 30 min. This precluded us from estimating total volume of moderate- to vigorous-intensity physical activity among children. No questions on the perceived availability of public recreation spaces, green space, and sports facilities, were available for this analysis. Although some of the survey items used in the parent project were sourced from validated instruments, others were developed specifically for the study. All survey items from previously validated instruments were culturally adapted and translated to Spanish for administration with the study population. Additionally, all survey items were pilot tested among community members of the target zip codes, to ensure cultural appropriateness and face validity. However, reliability testing and scale validation is not available for all included measures (23). The limited measures used in this study may partly explain why the health behavior models, and in particular the “change in child fruit and vegetable intake model,” were less robust in terms of the number of significant independent variables identified, as compared to the “change in BMI model” (which relied on objective weight and height data).

Another limitation of the two health behavior models is that neither of them fully captures the full sources of energy intake and expenditure. In terms of dietary intake, this analysis only explored a well-known component of a healthy diet: fruit and vegetable intake. There are many dietary sources necessary for characterizing a “healthy diet,” and the factors composing an “unhealthy diet” may be equally important for understanding longitudinal changes in BMI in children (4, 42).

Finally, even within this disadvantaged micro-area, we found that some factors at the environmental and community social norms levels, including the perceived lack of availability of neighborhood resources for physical activity, and perceived lack of community social norms favoring healthy eating, emerged as risk factors for decreases in physical activity and increases in BMI. These community-level variations may be a direct or indirect consequence of some of the environmental intervention strategies implemented in some of the study areas by the GAVA coalition (24). The dichotomous variable denoting geographic exposure status to these strategies, by virtue of residence in the target zip codes, was not found to be significantly associated to any of the studied dependent variables in this analysis. However, further analyses beyond the scope of this study should be conducted to quantify each participant's dose of exposure over time to these environmental strategies, to better understand how they may have influenced changes in their health behaviors and outcomes. In particular, it is possible that some of the associations that we found in this analysis between perceived community social norms and environmental factors and the outcomes of interest may be mediated or moderated by some of these community-led strategies to improve the food and built environment.

In spite of its limitations, our study also had many strengths. We focused on a geographic setting with a high-needs/high-risk population. Complete data were available for 91% of the child-parent dyads after 4 years of follow up. This, all within the context of a highly mobile, difficult-to-reach population (43–46). The data collection staff and instruments were bilingual, allowing us to collect high-quality, reliable data from a high proportion of Spanish-speaking-only parents and children, which are often excluded from studies (47). In this study, we sought to identify both factors associated with weight gain prevention as well as weigh loss over time, acknowledging that the positive determinants of one outcome may not necessarily be the inverse determinants of the other one (e.g., a factor associated with preventing weight gain may not necessarily be associated with promoting weight loss).

## Conclusions

In addition to the well-documented macro-level disparities associated with the disproportionate occurrence of childhood obesity and its behavioral determinants by race and income, our study revealed that among children residing in low-income, predominantly Hispanic neighborhoods in Austin, Texas, there are also important micro-level disparities. This study identified relevant factors at virtually all levels of the socio-ecological model, which may help in designing and testing contextually relevant obesity prevention interventions in these types of disadvantaged urban enclaves consisting of predominantly Hispanic residents. Additionally, through the characterization of *positive deviance* cases, our results revealed evidence that could potentially help in mitigating established unhealthy habits among high-need population groups.

## Data Availability Statement

The datasets used and/or analyzed during the current study are available from one of the authors, Dr. Nalini Ranjit: Nalini.ranjit@uth.tmc.edu.

## Ethics Statement

The studies involving human participants were reviewed and approved by Institutional Review Board of the University of Texas Health Science Center at Houston School of Public Health (HSC-SPH-13-0107). Written informed consent to participate in this study was provided by the participants' legal guardian/next of kin.

## Author Contributions

DS conceptualized the study presented in this manuscript (a secondary data analysis), conducted the analysis, prepared the tables, provided interpretation of the results, and led the drafting of the full manuscript. NR drafted some sections of the manuscript, contributed to interpretation of the results and data analysis decisions, and is the co-principal investigator of the parent study. AN coordinated all day to day activities for data collection of the parent study and critically reviewed the manuscript. NA was critically involved in data collection activities of the parent study, and critically reviewed the manuscript. AB led the team which conceptualized the parent study, helped conceptualize this secondary data study, contributed to interpretation of the results, and critically reviewed the manuscript.

### Conflict of Interest

The authors declare that the research was conducted in the absence of any commercial or financial relationships that could be construed as a potential conflict of interest.
